# LNA/DNA mixmer-based antisense oligonucleotides correct alternative splicing of the *SMN2* gene and restore SMN protein expression in type 1 SMA fibroblasts

**DOI:** 10.1038/s41598-017-03850-2

**Published:** 2017-06-16

**Authors:** Aleksander Touznik, Rika Maruyama, Kana Hosoki, Yusuke Echigoya, Toshifumi Yokota

**Affiliations:** 1 Department of Medical Genetics, University of Alberta Faculty of Medicine and Dentistry, Edmonton, Alberta Canada; 2 The Friends of Garrett Cumming Research & Muscular Dystrophy Canada HM Toupin Neurological Science Endowed Research Chair, Edmonton, Alberta Canada

## Abstract

Spinal muscular atrophy (SMA) is an autosomal recessive disorder affecting motor neurons, and is currently the most frequent genetic cause of infant mortality. SMA is caused by a loss-of-function mutation in the *survival motor neuron 1* (*SMN1*) gene. *SMN2* is an *SMN1* paralogue, but cannot compensate for the loss of *SMN1* since exon 7 in *SMN2* mRNA is excluded (spliced out) due to a single C-to-T nucleotide transition in the exon 7. One of the most promising strategies to treat SMA is antisense oligonucleotide (AON)-mediated therapy. AONs are utilized to block intronic splicing silencer number 1 (ISS-N1) on intron 7 of *SMN2*, which causes exon 7 inclusion of the mRNA and the recovery of the expression of functional SMN protein from the endogenous *SMN2* gene. We developed novel locked nucleic acid (LNA)-based antisense oligonucleotides (LNA/DNA mixmers), which efficiently induce exon 7 inclusion in *SMN2* and restore the SMN protein production in SMA patient fibroblasts. The mixmers are highly specific to the targeted sequence, and showed significantly higher efficacy than an all-LNA oligonucleotide with the equivalent sequence. These data suggest that use of LNA/DNA mixmer-based AONs may be an attractive therapeutic strategy to treat SMA.

## Introduction

Spinal muscular atrophy (SMA) is a recessive autosomal neuromuscular disorder characterized by the degradation of motor neurons within the anterior horn of the spinal cord, resulting in the progressive trunk and limb muscle paralysis^1^. SMA is currently the most common genetic cause of infant mortality^1^.

Most cases of SMA are caused by homozygous loss of the *survival of motor neuron 1* (*SMN1*) gene^2^. The *survival of motor neuron 2* (*SMN2*) gene is a modifier of the SMA phenotype and has the nearly identical sequence to *SMN1* with only a five-base pair difference^3, 4^. A C-to-T substitution in exon 7 of *SMN2* leads to the skipping of exon 7 in approximately 90% of *SMN2* transcripts^5^. The exon 7-skipped SMN2 protein cannot compensate for the SMN1 function because it is unstable and rapidly degraded. The remaining 10% of *SMN2* transcripts is not sufficient to rescue the SMA phenotype. However, a higher copy number of *SMN2* is associated with less severe clinical representations on average, even though it is not fully correlated^1, 6^.

Antisense therapy is currently one of the most promising strategies for treating SMA. Antisense oligonucleotides (AONs) are used to block intronic splicing silencer sites such as N1 (ISS-N1) on intron 7 of *SMN2*, which induces the inclusion of exon 7 and, consequently, leads to the recovery of functional SMN protein expression from the endogenous *SMN2* gene^7^. A 20-mer AON with phosphorothioate backbones and 2′-O-methyl (2′OMeP) modification was the first AON to show the efficacy of AONs *in vitro* for *SMN2* ^7^. Nusinersen/IONIS-SMN_Rx_ (Spinraza), an 18-mer AON with phosphorothioate backbones and 2′-O-methoxyethyl modification (MOE), increased the exon 7 inclusion and rescued SMA phenotypes *in vivo* in mouse models^8–11^. Following the completion of Phase I/II trials with encouraging data, nusinersen has recently been approved by the U.S. Food and Drug Administration (FDA) and became the first drug for the treatment of SMA^12–14^.

Although there is much hope for this drug, it has been known to present some complications since its pre-clinical testing. For example, nusinersen is not incorporated efficiently into certain cell types and tissues^8^. Repeated intrathecal injection is employed for its administration in the clinical trials^14^, because it cannot easily cross the blood-brain barrier^8^. Although intrathecal injections of nusinersen are considered to be safe, the typical side effects associated with lumbar puncture are induced in nearly one-third of treated patients in the clinical trial^15^. In addition, the manufacturer has announced that the treatment will cost $750,000 in the first year, and $375,000 every following year^16^. Development of a new and more affordable alternative would bring practical benefits for the patients.

To overcome these issues, the latest research is trying to identify better antisense chemistries. Administration of phosphorodiamidate morpholino oligomers (PMOs) targeting ISS-N1 by a single intracerebroventricular injection ameliorated the SMA symptom in the mouse models^17^. An 8-mer 2′OMeP AONs with PEG-282 and propyl modifications at the 5′ and 3′ ends also improved the phenotypes of SMA mouse models^18^. Recently, it has been demonstrated that peptide-conjugated PMOs (Pip6a-PMOs) were delivered to the central nervous system (CNS) by intravenous injections and rescued the phenotype of a severe SMA mouse model^19^. However, no clinical trial has been reported with these chemistries for SMA.

**Figure 1:**
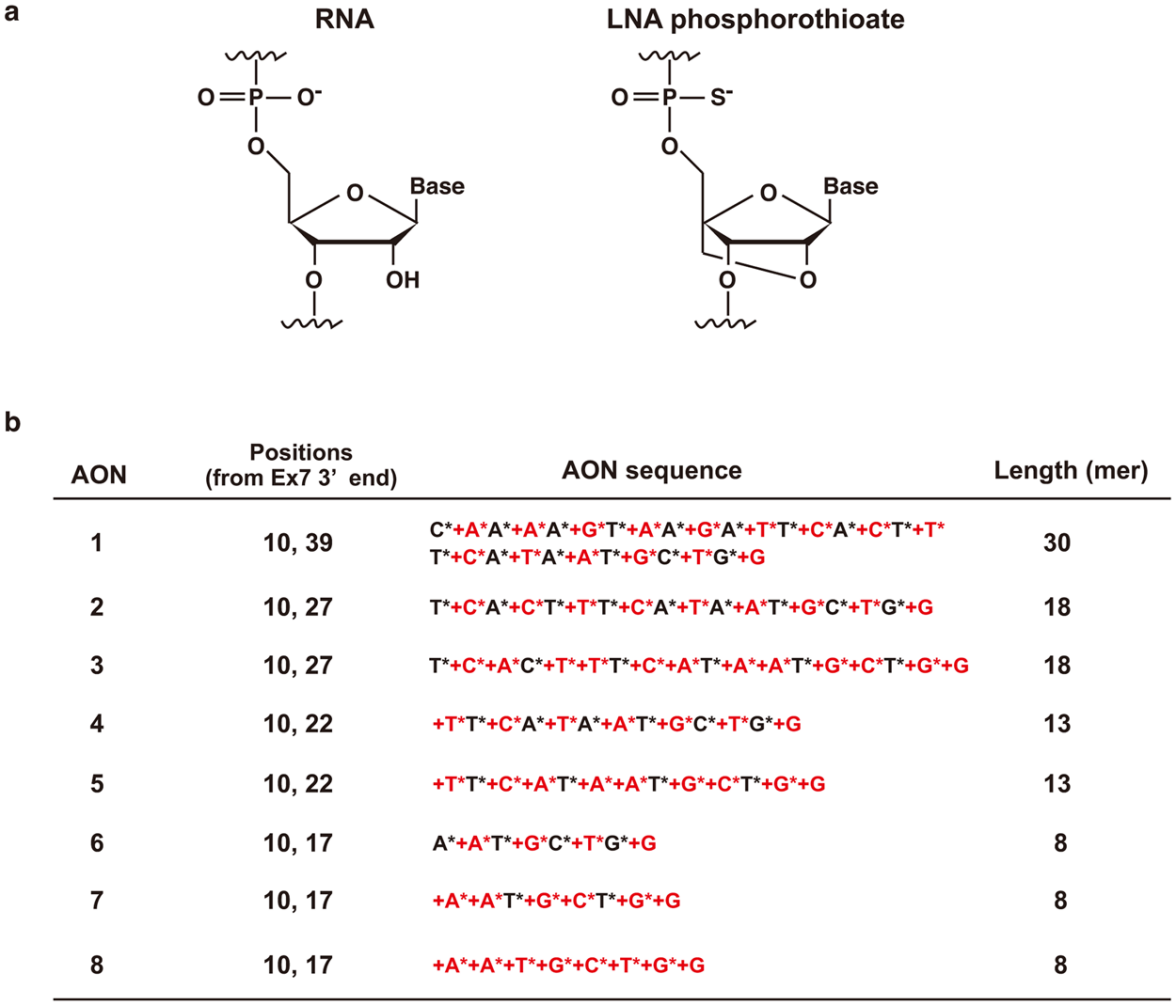
LNA structure and LNA/DNA mixmer sequences. (**a**) Chemical structures of RNA and phosphorothioated LNA. (**b**) LNA/DNA mixmer sequences targeting *SMN2* intron 7. DNA base: G, A, T, C. LNA base (red): +G, +A, +T, +C. Phosphorothioated DNA base: G*, A*, T*, C*. Phosphorothioated LNA base (red): +G*, +A*, +T*, +C*.

Locked nucleic acids (LNAs) are artificial nucleic acid analogs which contain a methylene bridge connecting the 2′-*O* with the 4′-*C* position in the furanose ring (Fig. 1a)^20, 21^. This modification makes them resistant to nucleases and increases affinity to complementary RNA sequences. The LNA chemistry has been used for gapmer AONs, single strand DNA oligonucleotides flanked by several LNA bases at the 5′ and 3′ ends^22^. Gapmer AONs bind targeted mRNAs and degrade them by activation of RNase H^22^. LNA/DNA mixmers (AONs composed of alternating LNA and DNA nucleotides) have been recently developed, which induce exon skipping in dystrophin mRNA (LNA-based splice-switching oligonucleotides) *in vitro* ^23^, or inhibit miRNA to protect the heart against pathological cardiac remodeling to improve the heart function (LNA-antimiR) *in vivo* ^24^. Miravirsen (AntimiR-122) is an LNA-antimiR that inhibits miR-122 to treat hepatitis C infection^25, 26^. MRG-106 (AntimiR-155) is another LNA-AntimiR and targets miR-155 for the therapy of cutaneous T cell lymphoma and mycosis fungoides^26^. The clinical trials of both LNA-antimiRs are currently ongoing^26^. One of the advantages of using LNA/DNA mixmers for treatment is that they have negatively charged backbones. This is expected to make delivery into cells more efficient than PMOs which are neutrally charged.

Here, we designed a series of LNA/DNA mixmers complementary to the ISS-N1, and evaluated their ability to induce exon inclusion in type I SMA patient fibroblasts. These AONs efficiently rescued the expression of SMN protein in the cultured cells. A single mismatch in AONs significantly decreased the exon inclusion efficacy, indicating that these AONs are highly specific to the target sequence. In addition, the LNA/DNA mixmer showed significantly higher efficacy than the all-LNA oligonucleotide with the equivalent sequence. Our results demonstrate that the LNA/DNA mixmers could be promising drug candidates suitable for *in vivo* studies to develop AON therapies to treat SMA.

## Results

**Figure 2:**
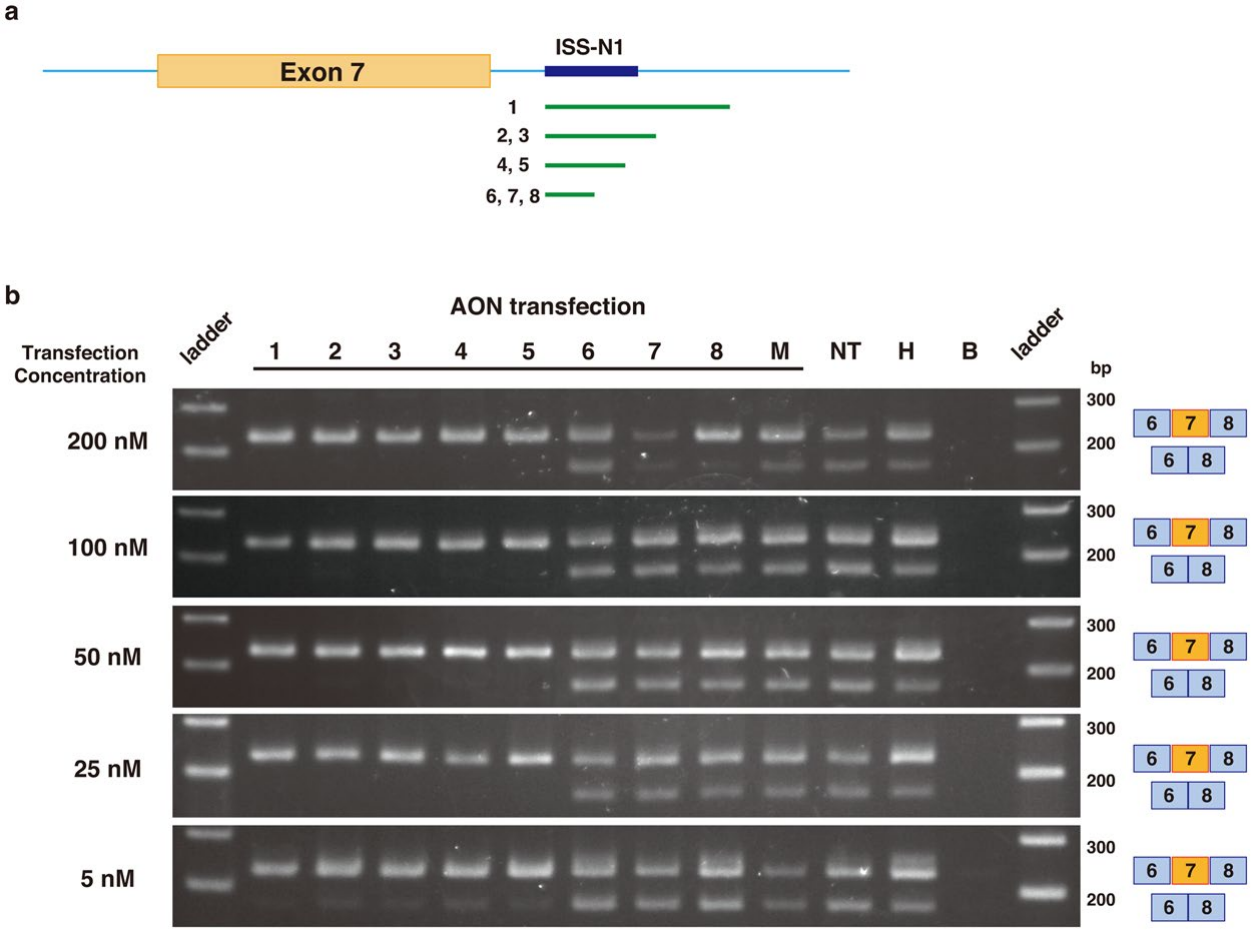
Newly designed AONs induce *SMN2* exon 7 inclusion. (**a**) Relative positions of newly designed AONs targeting ISS-N1 in intron 7 of *SMN2*. Green line: newly designed AONs (LNA/DNA mixmers). (**b**) The screen of AONs (LNA/DNA mixmers) by RT-PCR of *SMN2* in human SMA patient fibroblasts (GM03813). Transfection of AONs was performed at 200, 100, 50, and 5 nM. Transfection of at least 5 nM of AONs #1–5 induced the exon 7 inclusion. Top band: exon 7-included. Bottom band: exon 7-excluded. M: mock control AON, NT: non-treated, H: healthy cells, B: blank.

To utilize LNA/DNA mixmers for the antisense therapy for SMA, we designed eight antisense mixmers that target to the ISS-N1 in intron 7 of *SMN2* (Figs 1b and 2a). AON #1, #2, #4 and #6 are LNA-based 30-mer, 18-mer, 13-mer, and 8-mer oligonucleotides, respectively. They have a DNA substitution at every other nucleotide. AON #3, #5, and #7 are 18-mer, 13-mer, and 8-mer oligonucleotides, respectively. They are also composed of LNAs, with DNA being substituted for LNA at every third nucleotide position. All nucleotides of AON #8 are LNAs. All mixmers have fully modified phosphorothioated backbones to prevent degradation by nucleases. AON #2 and #3 (18-mer) have the same sequence as nusinersen (IONIS-SMN_RX_) and the PMOs previously published^8, 27^. Shimo *et al*. compared the efficacies of LNA/DNA mixmers whose lengths are between 6 mer to 23 mer, and 13 mer showed the best efficacy for exon skipping^23^. The 8 mer was the shortest LNA/DNA mixmer which induced efficient exon skipping^23^. As such, we employed 13 mer and 8 mer oligos in this study.

We first determined the optimal concentration of AONs to evaluate the efficacy of exon inclusion. AONs were transfected at various concentrations to type 1 SMA patient fibroblasts (Fig. 2b). The RT-PCR analysis using *SMN2* specific primers revealed that 5 nM or higher concentration of AONs #1–5 efficiently induced *SMN2* exon 7 inclusion in treated cells (Fig. 2b).

**Figure 3:**
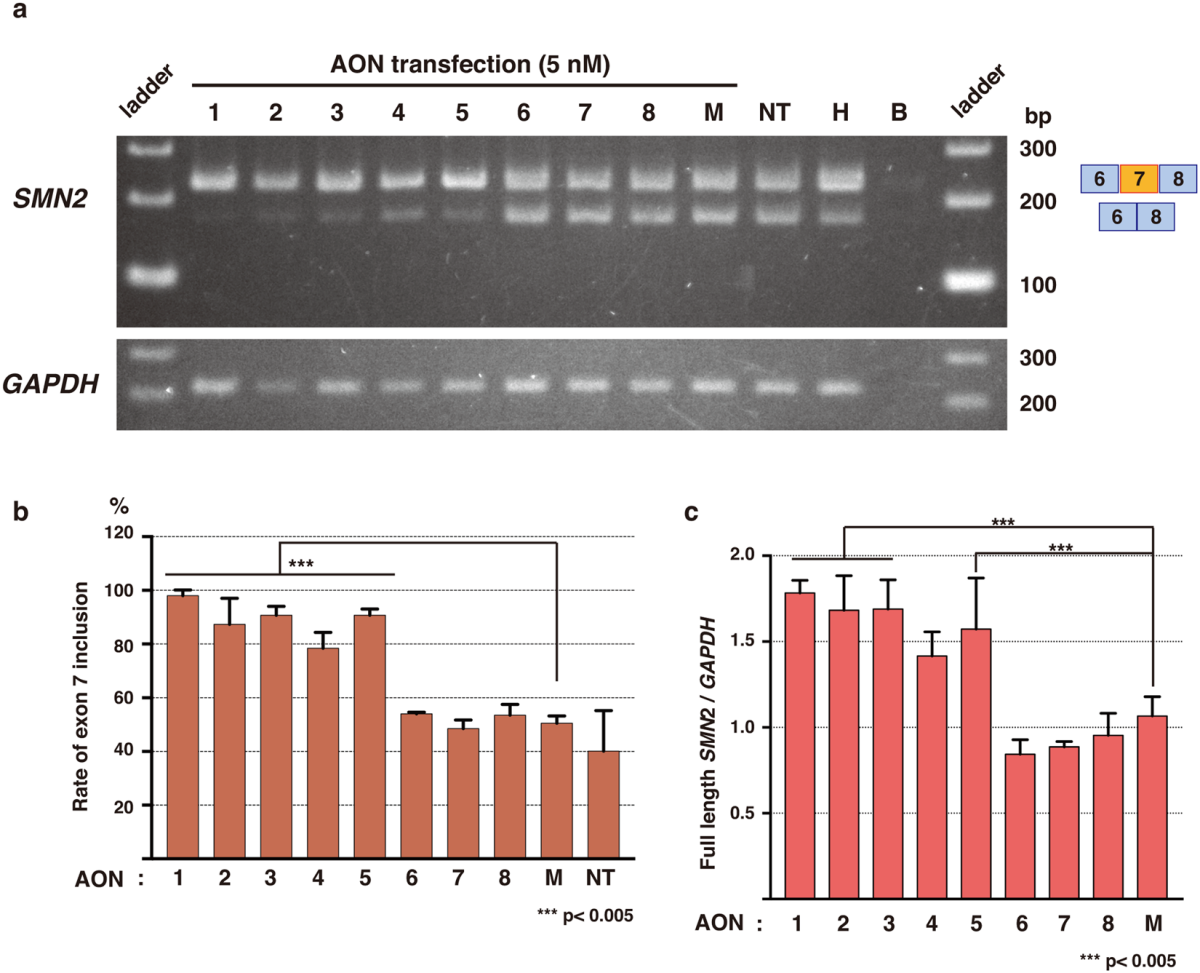
Efficacy of the novel LNA/DNA mixmers to induce *SMN2* exon 7 inclusion. 5 nM transfection of AONs #1-#5 induced *SMN2* exon 7 inclusion at a significantly higher rate than the mock AON transfection. (**a**) RT-PCR of *SMN2* and *GAPDH* in human SMA patient fibroblasts (GM03813). AONs #1–8 were transfected at 5 nM concentration. Top band: exon 7-included. Bottom band: exon 7-excluded. (**b**) Quantification of *SMN2* exon 7 inclusion in the AON-treated SMA fibroblasts by RT-PCR. (**c**) Relative expression of full-length *SMN2* to *GAPDH* measured by qPCR. The data were normalized to non-treated cells. Bars represent mean ± S.D. of three independent experiments. One-way ANOVA with Dunnett’s multiple comparison test. M: mock control AON, NT: non-treated, H: healthy cells, B: blank.

Next, we examined the efficacy of each AON. AONs were transfected into the SMA patient fibroblasts at 5 nM. Semi-quantitative RT-PCR using *SMN2* specific primers showed that the transfection of AONs #1–5 efficiently induced *SMN2* exon 7 inclusion in the patient cells (78–98% exon 7 inclusion, Fig. 3a,b). Transfection of AONs #6–8 at this dose did not show any significant difference from the control AON. Quantitative PCR (qPCR) also showed that the levels of full-length *SMN2* mRNA significantly elevated by the treatment of AONs #1–3 and #5, compared to the control (Fig. 3c).

**Figure 4:**
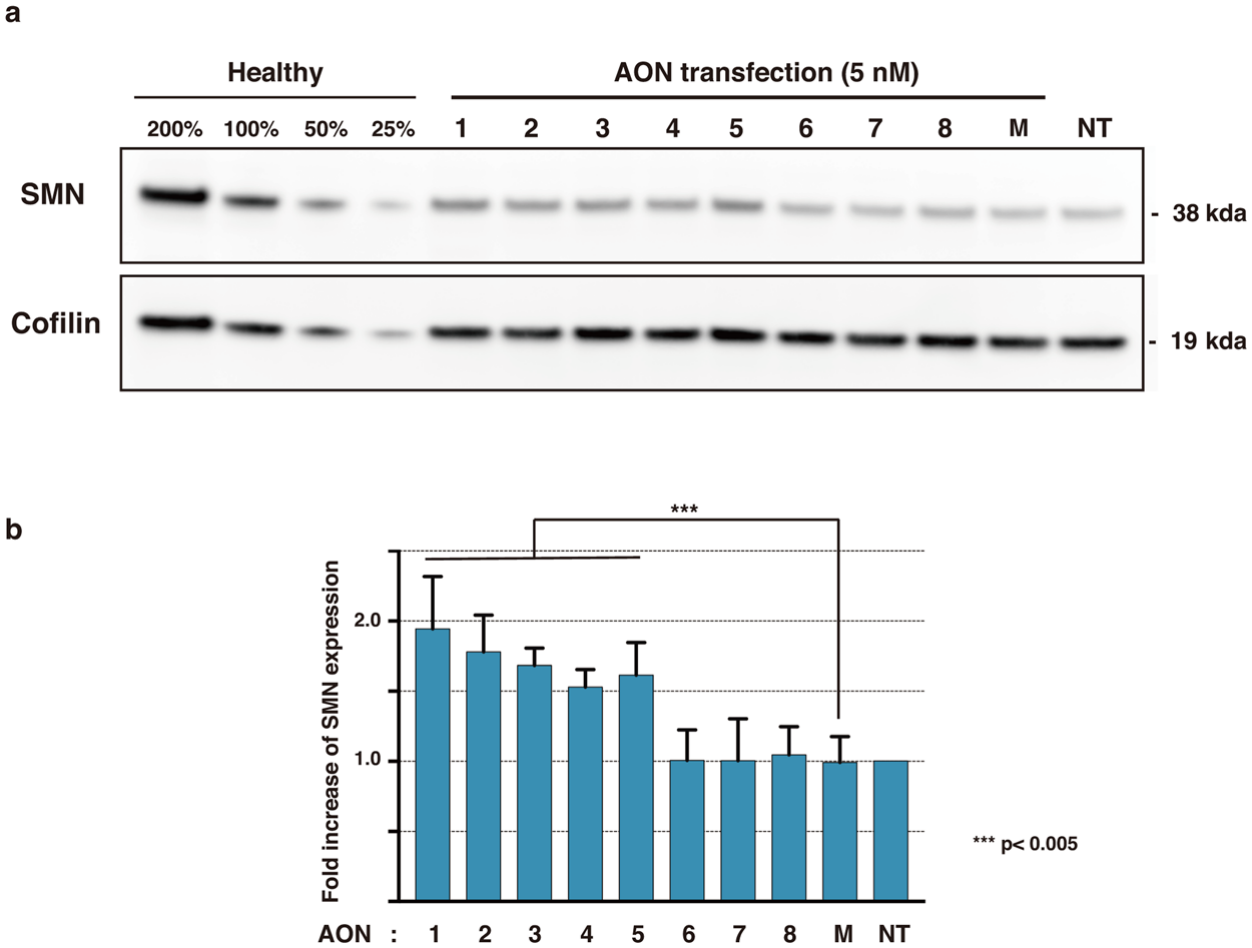
Transfection of the novel LNA/DNA mixmers increases the production of SMN protein. (**a**) Western blotting of SMN and Cofilin of healthy and AON-treated SMA patient fibroblasts (GM03813). (**b**) Fold increase in SMN expression of AON-treated SMA patient fibroblasts. 5 nM transfection of AON 1–5 significantly increased the production of SMN proteins. Cofilin was used as a loading control. The data was normalized to the ratio of SMN/Cofilin in non-treated SMA fibroblasts. Bars represent mean ± S.D. of four independent experiments. One-way ANOVA with Dunnett’s multiple comparison test. M: mock control AON, NT: non-treated.

In addition, Western blot protein analyses demonstrated that the levels of SMN protein significantly increased in the cells by transfection of AONs #1–5 (a 1.5–1.9-fold increase from the control, Fig. 4). The AONs #6–8 treatment did not change the levels of SMN protein in the cells. These data indicate that the LNA/DNA mixmers targeting ISS-N1 in *SMN2* intron 7 induce the exon 7 inclusion efficiently in the SMA patient cells.

**Figure 5:**
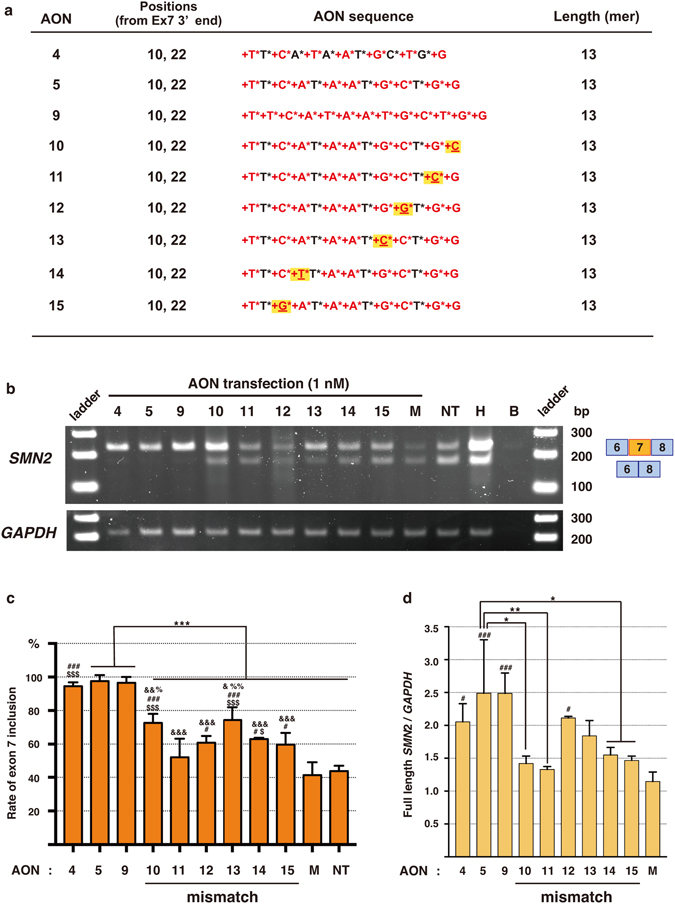
A single mismatch in the LNA/DNA mixmers abrogates the activity of them for exon 7 inclusion. (**a**) LNA/DNA mixmer sequences used for the experiment. The LNA/DNA mixmers #10–15 contain a single base mismatch at various locations along the sequence. AON #9 consists of 100% LNA. DNA base: G, A, T, C. LNA base (red): +G, +A, +T, +C. Phosphorothioated DNA base: G*, A*, T*, C*. Phosphorothioated LNA base (red): +G*, +A*, +T*, +C*. Yellow highlighted: mismatch. (**b**) RT-PCR of *SMN2* and *GAPDH* in human SMA patient fibroblasts (GM03813). AONs were transfected at 1 nM concentration. Top band: exon 7-included. Bottom band: exon 7-excluded. (**c**) Quantification of *SMN2* exon 7 inclusion in AON-treated SMA fibroblasts by RT-PCR. ***p < 0.005. ^#^Significant difference with the mock (^#^p < 0.05, ^##^p < 0.01, ^###^p < 0.005). ^$^Significant difference with the non-treated (^$^p < 0.05, ^$$^p < 0.01, ^$$$^p < 0.005). ^&^Significant difference with AON 4 (^&^p < 0.05, ^&&^p < 0.01, ^&&&^p < 0.005). ^%^Significant difference with AON 11 (^%^p < 0.05, ^%%^p < 0.01). (**d**) Relative expression of full-length *SMN2* to *GAPDH* measured by qPCR. The data were normalized to non-treated cells. *p < 0.05, **p < 0.01, ***p < 0.005. ^#^Significant difference with the mock (^#^p < 0.05, ^##^p < 0.01, ^###^p < 0.005). Bars represent mean ± S.D. of three independent experiments. One-way ANOVA with a Tukey’s multiple comparison test. M: mock control AON, NT: non-treated, H: healthy cells, B: blank.

To determine the specificity of the LNA/DNA mixmers, we designed AONs containing a single mismatch in AON #5 (Fig. 5a). We compared the efficacy of AON #5 with AONs of the same sequence but including a single mismatch (AONs #10–15) by the semi-quantitative RT-PCR using *SMN2* specific primers. The results indicated that all AONs containing a single mismatch showed significantly lower efficiency of exon 7 inclusion compared to AON #5 (Fig. 5b,c). Quantitative PCR (qPCR) also confirmed that the amount of full-length *SMN2* mRNA did not increase by the treatment of most AONs with a single mismatch, AON #10, #11, #13, #14 and #15 (Fig. 5d). The data suggest that these LNA/DNA mixmers are highly specific to the targeted sequence, with a single mismatch leading to a significant loss of binding affinity between the mixmers and the targeted mRNAs.

**Figure 6:**
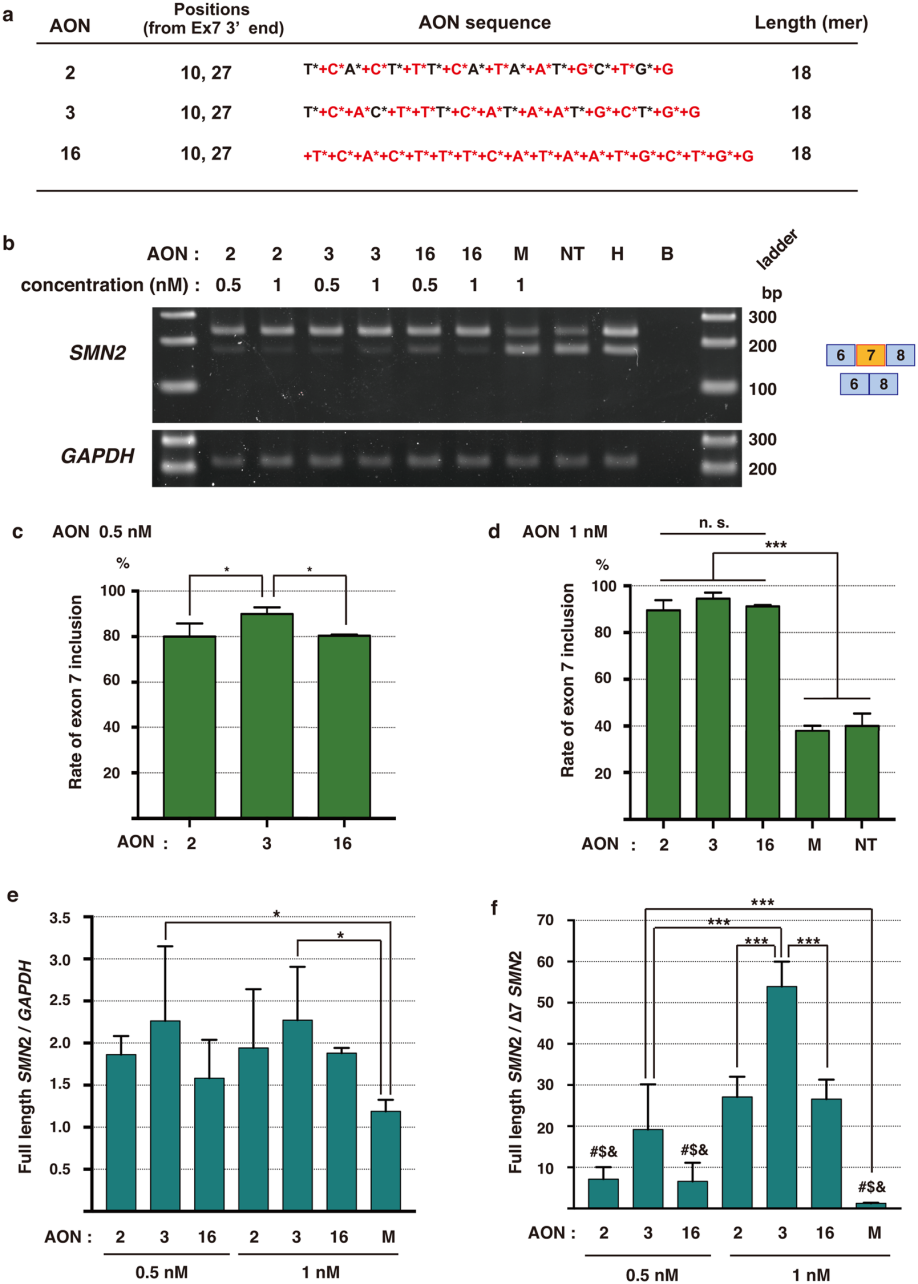
LNA-to-DNA replacement improves exon 7 inclusion activity. (**a**) AON sequences used for the experiment. AON #16 consists of 100% LNA. DNA base: G, A, T, C. LNA base (red): +G, +A, +T, +C. Phosphorothioated DNA base: G*, A*, T*, C*. Phosphorothioated LNA base (red): +G*, +A*, +T*, +C*. (**b**) RT-PCR of *SMN2* and GAPDH in human SMA patient fibroblasts (GM03813). AONs were transfected at 0.5 or 1 nM concentration. Top band: exon 7-included. Bottom band: exon 7-excluded. (**c**) Quantification of *SMN2* exon 7 inclusion in AON-treated SMA fibroblasts by RT-PCR. AONs were transfected at 0.5 nM concentration. *p < 0.05. (**d**) Quantification of *SMN2* exon 7 inclusion in AON-treated SMA fibroblasts by RT-PCR. AONs were transfected at 1 nM concentration. ***p < 0.005. (**e**) Relative expression of full-length *SMN2* to *GAPDH* measured by qPCR. The data was normalized to non-treated cells. *p < 0.05. (**f**) Relative expression of full-length *SMN2* to ∆7 *SMN2* measured by qPCR. The data were normalized to non-treated cells. ***p < 0.005. ^#$&^Significant difference with AON #2, #3, and #16 (1 nM treatment, p < 0.005). Bars represent mean ± S.D. of three independent experiments. One-way ANOVA with a Tukey’s multiple comparison test. n.s.: not significant. M: mock control AON, NT: non-treated, H: healthy cells, B: blank.

Moreover, we compared the efficacies between LNA/DNA mixmers and all-LNA oligonucleotides and examined whether LNA-to-DNA substitution could improve the efficacy. When the 13 mer AONs were transfected into the cells at 1 nM, the efficiency of exon inclusion between the mixmers (AONs #4 and #5) and the all-LNA oligonucleotide with the equivalent sequence (AON #9) was not significantly different (Fig. 5b–d). However, when the 18-mer mixmers (AONs #2 and #3) and the all-LNA oligonucleotide with the equivalent sequence (AON #16) were transfected at 0.5 nM, semi-quantitative RT-PCR showed that one of the mixmers (AON #3) had a significantly higher efficacy than the all-LNA oligonucleotide (AON #16) (Fig. 6c). In addition, qPCR revealed that only AON #3 treatment significantly increased the amount of full-length *SMN2* compared to the control at 0.5 nM or 1 nM transfection (Fig. 6e). The ratio of full-length to exon 7-deleted *SMN2* of AON #3 was significantly larger than that of the all-LNA oligonucleotide (AON #16) at 1 nM transfection (Fig. 6f). These results suggest that the LNA/DNA mixmer could have better efficacies than all-LNA oligonucleotides with an equivalent sequence for *SMN2* exon inclusion.

## Discussion

In this study, we designed a series of LNA-based splice-switching oligonucleotides (SSOs) targeting human *SMN2* ISS-N1 in intron 7, and examined their ability to induce exon inclusion. We demonstrated that five LNA/DNA mixmers we designed efficiently induce exon inclusion and recover the expression of SMN protein in type I SMA patient fibroblasts (Figs 3 and 4). A single mismatch significantly decreased the ability to induce exon inclusion, suggesting that these LNA/DNA mixmers are highly specific to the targeted sequence (Fig. 5). This is the first report showing that LNA/DNA mixmers induce efficient exon inclusion. It might, therefore, be an attractive therapeutic strategy to treat SMA.

The results of the RT-PCRs and the qPCR indicate that transfection with the mixmers at 5 nM induces nearly 100% exon 7 inclusion in SMA fibroblast cells (Figs 2 and 3), whereas 2′MOE oligos (nusinersen) were reported to induce the exon inclusion at 100 nM *in vitro* ^28^. The effective concentration for the mixmer transfection is also lower than that of the PMO transfection (100 nM)^27^. Additionally, the results indicate that the 13-mer AONs are sufficient to target ISS-N1 and induce the exon inclusion whereas the 8-mer AONs are not, corresponding to the previously published data with 2′OMeP AONs^18, 29^. The ISS-N1 has two hnRNP A1 binding sites^7, 29^. The 8-mer AONs block only one of these sites, while the 13-mer AONs cover the one and more than half of the other sites. These indicate that both hnRNP A1 binding sites should be targeted by AONs to induce exon inclusion.

LNA, or 2′-O,4′-C-methylene-bridged nucleic acid (2′,4′-BNA), is an artificial nucleic acid developed independently by Wengel’s group and Obika’s group in the late 1990s (Fig. 1a)^20, 21, 30^. Although LNAs have been used for various gene silencing techniques, e.g. antisense gapmer, short interfering RNA, blocking of microRNA, and triplex-forming oligonucleotides^22, 24, 31, 32^, it has not been used as an SSO until recently. Singh *et al*. showed a 14-mer all-LNA oligonucleotide targeting ISS-N1 induced exon inclusion of *SMN2* ^33^. In addition, it has been reported that alternating LNA/deoxyribose oligonucleotides, or LNA/DNA mixmers, efficiently induce exon skipping *in vitro* ^23, 34^. In contrast to Singh *et al*.^33^, SSOs only containing LNAs showed very low ability to induce exon skipping in the *DMD* exon 58^23^. Shimo *et al*. and Yamamoto *et al*. hypothesized that it is probably because LNA-only-SSOs possess extremely high binding affinity to mRNAs and that the high binding affinities of LNAs reduce the dissociation rate from targeted mRNAs and prevent the efficient turnover of SSOs^23, 35^. Meanwhile, our data indicated that the proportion and the positions of LNA and DNA in mixmers affected the exon inclusion efficiency. Interestingly, AON #3, which is composed of LNAs with DNA being substituted for LNA at every third nucleotide position, was more effective than AON #2 (DNA substitution at every other nucleotide) and AON #16 (all-LNA oligonucleotide) (Fig. 6). It suggests that the ratio and the position of LNAs and DNAs are critical factors to determine the efficacies of LNA/DNA mixmers.

AONs containing LNAs cause hepatotoxicity in some cases because the strong binding affinity to mRNAs induces off-target effects to pre-mRNAs, particularly in the liver^36^. This toxicity is reported to be a sequence-specific issue rather than an issue associated with the LNA chemistry^36^. According to NCBI BLAST, there is no human mRNA which includes entire complementary sequences of AON #1, #2 and #3. However, AON #4 and #5 (13 mer) are identical to part of complementary sequences of dystrophin-related protein 2 (DRP2) and peroxisome proliferator activated receptor alpha (PPARA) mRNAs. Considering this and the result of Fig. 6, AON #3 might be the most suitable AONs for *SMN2* exon inclusion among the mixmers we tested. The potential toxicity of each mixmer should be carefully evaluated *in vivo* before proceeding to clinical trials.

Our study has shown that the LNA/DNA mixmers we developed have very high efficacy *in vitro*. Administration of mixmers could have a significant effect *in vivo*, even with lower doses. From a therapeutic point of view, administration of lower concentrations and minimizing administration frequency would be desirable because of safety concerns and the cost of the treatment. Therefore, LNA/DNA mixmers are one of the promising drug candidates suitable to develop antisense oligonucleotide therapies to treat SMA.

## Methods

### AONs (LNA/DNA mixmers)

All LNA/DNA mixmers were synthesized by Exiqon. Each mixmer sequence was examined by BLAST software to check for any potential negative off-target effects. The mock AON sequence is +T*A* +A*C* +A*C* +G*T* +C*T* +A*T* +A*C* +G*C* +C*C* +A. Phosphorothioated DNA base: G*, A*, T*, C*. Phosphorothioated LNA base: +G*, +A*, +T*, +C*.

### Cells and AON transfection

The SMA patient fibroblasts (GM03813, Coriell NIGMS human genetic cell repository) were obtained from a male patient diagnosed with type I SMA. The cells have a homozygous deletion of exons 7 and 8 in *SMN1* and have two copies of the *SMN2* gene. Healthy fibroblasts (GM23815, Coriell NIGMS human genetic cell repository) were used as a control. AON transfection was performed with Lipofectamine RNAiMAX (Invitrogen) in OptiMEM I serum-reduced media (Gibco) for 48 hours.

### RT-PCR

Total RNA was extracted using TRIzol Reagent (Invitrogen). RT-PCR was performed using SuperScript III One-Step RT-PCR System with Platinum Taq High Fidelity (Invitrogen). The primers were designed to detect only *SMN2* sequences (not to detect *SMN1*) in the SMA cell line, as the fibroblast cells from this patient have a deletion mutation of exons 7 and 8 in *SMN1*. The *SMN2* primer sequences were 5′-CTGCCTCCATTTCCTTCTG-3′ (Forward) in exon 6 and 5′-TGGTGTCATTTAGTGCTGCTC-3′ (Reverse) in exon 8. The *GAPDH* primer sequences were 5′-TCCCTGAGCTGAACGGGAAG-3′ (Forward) and 5′-GGAGGAGTTTGGTCGCTGT-3′ (Reverse). The cDNA was synthesized for 5 min at 50 °C. The *SMN2* exon 6–8 was amplified with 30 PCR cycles (94 °C for 15 sec, 60 °C for 30 sec, 68 °C for 25 sec). The GAPDH was amplified with 20 PCR cycles (94 °C for 15 sec, 60 °C for 30 sec, 68 °C for 20 sec).

### qPCR

Total RNA was extracted using TRIzol Reagent (Invitrogen), and cDNA was generated using SuperScript III Reverse Transcriptase (ThermoFisher). The qPCR reaction was performed by SsoAdvanced Universal SYBR Green Supermix (Bio-Rad) and QuantStudio3 real-time PCR system (Applied Biosystems). The full-length *SMN2* transcripts were amplified using the primer set: (forward, 5′-GCTATCATACTGGCTATTATATGGGTTTT-3′; reverse, 5′-CTCTATGCCAGCATTTCTCCTTAAT-3′). The ∆7 *SMN2* transcripts were amplified using the primer set: (forward, 5′-TCTGGACCACCAATAATTCCCC-3′; reverse, 5′-ATGCCAGCATTTCCATATAATAGCC-3′). The expression of *GAPDH* was measured using the primer set (forward, 5′-GCAAATTCCATGGCACCGT-3′; reverse, 5′-AGGGATCTCGCTCCTGGAA-3′). The relative expression of full-length *SMN2* to *GAPDH* or ∆7 *SMN2* was calculated, and normalized to the non-treated cells with the ∆∆Ct algorithm.

### Western Blotting

Whole cell protein was collected using Pierce RIPA lysis buffer (Thermo Scientific) with 1x Roche cOmplete protease inhibitor. Western blotting was performed, as described^37^. Five μg of total protein was run per well in NuPAGE Novex 4–12% Bis-Tris Midi Protein Gels (Life Technologies). The mouse purified anti-SMN antibody (BD Biosciences) and the rabbit anti-Cofilin antibody (D3F9, Cell Signaling) were used as primary antibodies (Both were diluted to 1: 10,000, incubated for 1 hour at room temperature). HRP-conjugated goat anti-mouse IgG (H + L) (Bio-Rad) and HRP-conjugated goat anti-rabbit IgG (H + L) (Bio-Rad) were used as secondary antibodies (Both were diluted to 1:10,000). The bands were detected using Amersham ECL Select Western blotting detection kit (GE Healthcare).

### Quantification and Statistical Analysis

Image J software (NIH) was used to quantify the band intensity. For RT-PCR of *SMN2*, the intensity of full-length bands and ∆7 bands were measured. The rate of exon 7 inclusion was calculated by the intensity value of (full-length)/(full-length + ∆7 *SMN2*). The RT-PCR, the qPCR, and the Western blotting data were analyzed by one-way repeated measures ANOVA with Tukey’s or Dunnett’s multiple comparison post hoc analysis by GraphPad Prism 7 (GraphPad software).
